# Enhancing physician scientists’ skills in Geographic Information Systems: insights from an interactive workshop

**DOI:** 10.1080/19475683.2025.2548205

**Published:** 2025-08-18

**Authors:** Muktar H. Aliyu, Ibraheem M. Karaye, Chelsea van Wyk, Aishatu L. Adamu, Fatimah I. Tsiga-Ahmed, Hafsah B. El Yakubu, Rukayya S. Alkassim, Baba M. Musa, Mahmoud U. Sani, C. William Wester

**Affiliations:** a Vanderbilt Institute for Global Health, Vanderbilt University Medical Center, Nashville, USA; b Department of Population Health, Hofstra University, Hempstead, USA; c Department of Anesthesiology, Montefiore Medical Center, Bronx, USA; d Department of Community Medicine and Department of Medicine, Bayero University Kano & Aminu Kano Teaching Hospital, Kano, Nigeria; e Africa Center of Excellence for Population Health and Policy, Bayero University, Kano, Nigeria; f Department of Medicine, Division of Infectious Diseases, Vanderbilt University Medical Center, Nashville, USA

**Keywords:** Geographic Information Systems, capacity building, global health, physician scientists, low- and middle-income countries

## Abstract

Geographic Information Systems (GIS) have become essential in health sciences for analysing and visualizing health-related spatio-temporal data. This report details the outcomes of a week-long interactive GIS workshop held in Kano, Nigeria, and organized by the Vanderbilt-Nigeria Building Research Capacity in HIV and NCDs (V-BRCH) training programme. The workshop aimed to enhance GIS knowledge and confidence among early-career physician scientists. Thirty-three participants were trained in core GIS competencies, including software selection, data visualization and spatial analysis using Quantum GIS (QGIS). Pre- and post-workshop surveys assessed participants’ knowledge and confidence levels across various GIS topics and competency areas. There was a significant improvement in self-reported participant knowledge across all GIS topic areas evaluated, with the highest percentage gains in geocoding health data (149%) and using QGIS software (135%). The percentage increase in post-workshop confidence was greatest for importing spatial data into QGIS (153%), navigating the QGIS interface (150%) and mapping public health data (150%). Participants rated the workshop highly (4.7/5, 1 = ‘poor’ and 5 = ‘excellent’). Recommendations for course improvement included extending the duration of the workshop, using local data in exercises and employing more visual aids. These findings suggest that GIS training opportunities can be beneficial in building GIS knowledge and enhancing the skills of physician scientists in similar low- and middle-income settings.

## 1. Introduction

The application of Geographic Information Systems (GIS) in the health sciences is increasingly recognized as a valuable tool in processing, analysing and visualizing complex spatio-temporal data patterns and trends related to health outcomes and ecological contexts (Cromley and McLafferty 2012; Kistemann, Dangendorf, and Schweikart 2002; Ruankaew 2005; Xu et al. 2024). GIS provides useful multidisciplinary evidence and user-friendly information that informs decision making across various fields, including public health, environmental health, medicine, infectious diseases and epidemiology, among many others (Nykiforuk and Flaman 2011).

Efforts to build capacity in GIScience are relatively new but expanding rapidly. Consistent with the multidisciplinary nature of GIScience, previous workshops covered diverse topics and targeted various groups. Examples include a two-week population/GIS workshop for geography faculty and researchers in Cambodia, an introductory GIS workshop for geospatial librarians to explore and document geographic patterns in racially biased policing practices in Colorado, and a five-day U.S. National Cancer Institute-supported initiative that brought together experts and stakeholders to address current issues in GIScience and cancer control (Jokilosch and Boone 2003; Pickle et al. 2006; Ranganath 2022). However, most of these workshops were not held in low- and middle-income countries (LMICs), and to our knowledge, none were solely devoted to training physician scientists. Reflecting on earlier calls to incorporate GIS training into public health college curricula (Clarke, Sl, and Tempalski 1996), there is a pressing need for physician scientists, especially in resource-constrained settings like Nigeria, to acquire skills in GIS. Such training will enable them to apply advanced, multidisciplinary computer-based techniques in their research, ultimately enhancing the quality and impact of their scientific work. Physician scientists can benefit from basic GIS skills to enhance patient care and public health decision-making, such as the ability to create and interpret maps, perform spatial analysis and integrate diverse health-related data. Physicians can apply GIS skills to tracking the geographic spread of infectious diseases, assessing healthcare access in underserved regions and identifying regional trends and patterns in the distribution of diseases. Such data can then be employed for public health planning, resource allocation, more effective healthcare delivery and targeted disease prevention strategies.

The Vanderbilt-Nigeria Building Research Capacity in HIV and NCDs programme (V-BRCH), supported by the Fogarty International Center (FIC) of the National Institutes of Health (NIH), aims to empower Nigerian physician scientists to conduct high-quality clinical research on HIV-associated NCDs (Aliyu et al. 2021). V-BRCH provides formal training to early-stage physician scientists to enhance their research skills via a blend of short- and medium-term educational opportunities, including interactive workshops held in Nigeria. These workshops address specific research topics and skills identified as important gap areas requiring enhancement. One such workshop was conducted in June 2024 and introduced participants to fundamental GIS concepts. The training covered practical skills such as selecting and using GIS software, visualizing health data, preparing and geocoding health event data, designing and implementing mapping, and performing basic spatial analysis with a publicly available open-source GIS program.

The purpose of this paper is to describe the changes in the workshop participants’ self-perceived knowledge and confidence in GIS-related topics and skills, as well as their perspectives on the effectiveness of the workshop and suggestions for future improvements to the training.

## 2. Materials and methods

The GIS workshop took place over five days in June 2024 at the conference centre of the Africa Centre of Excellence in Population Health and Policy, located within Aminu Kano Teaching Hospital (AKTH), in Kano, Nigeria. The workshop was widely promoted through the V-BRCH webpage and across AKTH and its affiliated academic institution, Bayero University, Kano (BUK), using emails and social media platforms.

### 2.1. Participants

Potential participants were invited to apply for the workshop through a REDCap application (Harris et al. 2009). As part of the application process, they were required to submit background information and a personal statement detailing their motivation to attend, which helped tailor participant selection and guide instruction. Applicants were also required to obtain approval from their supervisors to commit to the full five days of the workshop. Completed applications were reviewed by the three principal investigators and a senior program manager. Priority was given to early-stage physician scientists (instructor or assistant professor level) affiliated with AKTH/BUK, as well as current trainees or alumni of other NIH/Fogarty-funded training programmes at AKTH with interest in learning GIS concepts, strategies and design principles.

### 2.2. Objectives

The workshop objectives, topics and content were developed collaboratively, incorporating input from both Nigerian partners and US-based colleagues. The objectives of the workshop were as follows: 1) to help participants grasp core GIS concepts and acquire practical skills in health data visualization and analysis using a publicly available open-source GIS program. The adoption of open-source GIS software like Quantum GIS (QGIS) in LMIC settings offers significant advantages, including enhanced affordability, increased accessibility and reduced reliance on commercial vendors; 2) to enable participants to select and use GIS software, visualize health data, prepare and geocode health event data, design and implement mapping and perform basic spatial analyses; and 3) to guide participants to complete a hands-on mini GIS project that reinforces the application of the knowledge and skills acquired during the workshop.

### 2.3. Specific training activities

The workshop was conducted through a combination of didactic presentations, hands-on small group activities and interactive discussions. We used a learner-adaptive format, modifying training content and sequence according to participants’ performance, learning needs and responses (Table 1). The first day of the workshop focused on GIScience and spatial epidemiology and featured a didactic overview of GIS, applications of GIS and spatial epidemiology, and different types of GIS software, including an introduction to Quantum GIS (QGIS, https://qgis.org/) and its features. Participants also installed QGIS software on their laptops. Day 2 included lectures on the fundamentals of spatial data, covering data representation and structure, map scale and projections, as well as different map types. Hands-on sessions introduced participants to the QGIS interface, including navigating the interface, loading data layers and managing and visualizing different types of spatial data. Day 3 comprised both didactic and skill-based sessions on geospatial data acquisition, manipulation and area-based measures. Small group activities focused on preparing and geocoding health event data. Participants were introduced to advanced data visualization techniques, such as thematic mapping and label placement on Day 4, with sufficient time allocated for hands-on practice in mapping and map design. Day 5 included introductory lectures on geospatial data analysis and modelling, with participants performing basic spatial analysis tasks, such as buffer analysis and spatial joins in QGIS. The complete workshop schedule is available at https://www.vumc.org/v-brch/gis-workshop. The instructors for the workshop included a consultant with extensive experience in public health GIS applications, two support faculty from Bayero University Kano (BUK) and the V-BRCH multiple principal investigators.

### 2.4. Evaluation

Participants were notified of acceptance via email, which included a secure REDCap link to a structured pre-workshop survey. This baseline survey captured demographic information and assessed participants’ self-perceived knowledge and confidence in specific GIS topics and skills/competencies using a Likert scale (1 = no knowledge/not confident at all to 4 = advanced knowledge/very confident). The same questions were included in the post-workshop survey for comparison. These Likert scale ratings for self-perceived knowledge and confidence were then transformed to an ordinal scale: 4 = 100, 3 = 75, 2 = 50 and 1 = 25. Average scores for knowledge and confidence in these specific topics and competencies were calculated using these values. The difference between the pre- and post-workshop average rankings for self-perceived knowledge and confidence indicated the extent of change in these variables. The percentage increase in knowledge and confidence level was then computed by dividing this difference by the pre-workshop average.

The post-workshop assessment also gathered participants’ overall impressions of the instructor’s subject matter expertise, presentation quality, discussion time and perceived usefulness of the programme. Participants provided open-ended feedback on what they liked about the workshop, what they would change and suggestions for future topics. To analyse this feedback, we used a word cloud generator to identify the most frequently mentioned words and phrases, highlighting key areas for consideration in future workshops. Similar responses that referred to the same topic, even if phrased differently, were grouped and tallied.

The design of both the pre- and post-workshop surveys was guided by the Kirkpatrick Four-Level Training Evaluation Model (Kirkpatrick and Kirkpatrick 2006), with survey questions adapted from those used in previously published workshop papers (Aliyu, Sani, Ingles, Tsiga-Ahmed, Musa, Ahonkhai, et al. 2022; Aliyu, Sani, Ingles, Tsiga-Ahmed, Musa, Byers, et al. 2022; Gibas et al. 2024; Shepherd et al. 2023). A programme manager summarized the evaluation results. Ethical approval for the programme was obtained from the AKTH Ethics Review Committee. All responses were treated as confidential.

## 3. Results

Workshop participants were surveyed to evaluate their self-perceived knowledge and confidence in various topics and skills before and after the workshop, and to gather their feedback on the programme. The pre-workshop survey responses from all 33 participants (100%) were complete. Of the post-workshop surveys, 29 responses were complete (response rate: 87.9%), while two surveys were incomplete and two were not started. Out of 33 participants, all but 3 were faculty members from AKTH or BUK. The majority of participants were male (23/33, 69.7%) and early-career faculty members at the instructor or assistant professor level (27/33, 81.8%) (Table 2). The most common specialities were medicine (12/33, 36.4%), surgery (7/33, 21.2%), laboratory sciences (6/33, 18.2%) and public health (5/33, 15.1%). Of the 33 pre-workshop respondents, only 5 had received prior training in GIScience or spatial epidemiology. Applicants mentioned improved ability to address complex research questions and being able to use GIS to support evidence-based health policy and tackle important public health issues as motivating factors to attending the workshop.

Among all workshop participants, the average pre-workshop knowledge level across all GIS topic areas assessed was 38.3 (standard deviation, SD = 4.4). Most respondents rated their knowledge of specific GIS topics between 1 (‘no knowledge’) and 2 (‘little knowledge’) on a scale of 1 to 4. On the post-workshop survey, the average knowledge level among all participants across all GIS topic areas was 82.6 (SD = 3.0), indicating that most respondents rated their knowledge of specific topics in GIS between 3 (‘some knowledge’) and 4 (‘advanced knowledge’). There was a significant improvement in participant-reported knowledge in all GIS topic areas measured, with post-workshop knowledge averages greater than 80 in most topic areas (Table 3). The percentage increase in participant reported knowledge was greatest for ‘geocoding public health data’ (149%) and QGIS software (135%) and least for ‘visualizing health data’ (79%).

Among all workshop participants, the average pre-workshop confidence level across all GIS competencies assessed was 35.9 (SD = 2.3), while the average post-workshop confidence level among all participants was 84.0 (SD = 5.5). Prior to the workshop, respondents rated their confidence level in GIS competencies between 1 (not confident at all) and 2 (‘a little confident’). The pre-workshop self-reported scores showed that participants had the highest average knowledge and confidence in ‘visualizing health data’. Post-workshop average ratings all ranged between 3 (‘somewhat confident’) and 4 (‘very confident’) (Table 4). The increase in post-workshop confidence averages for all competencies was greatest for importing spatial data into QGIS (153%), navigating the QGIS interface (150%) and mapping public health data into QGIS (150%). ‘Visualizing health data’ showed the lowest improvement in reported confidence, but this change was still more than a 100% improvement.

We used the post-workshop surveys to ascertain feedback regarding the workshop and the instructors (Table 5). On a rating scale of 1 to 5, with 1 being ‘poor’ and 5 being ‘excellent’ participants provided an average overall rating for the course of 4.7 out of 5, while the overall effectiveness of the instructors was rated at 4.9 out of 5. When asked to rate the difficulty of the course on a scale of 1 to 100, with 1 being ‘much too easy’ and 100 being ‘much too hard’ participants on average rated the course at 47.1 (SD = 23.4). Participants also found the course to be highly organized with an average rating of 84.6 (SD = 18.1) on a scale of 1 to 100 (1 = ‘not at all organized’ and 100 = ‘extremely well organized’). The ease of learning information during the course was rated by participants as 78.9 (SD = 16.2) (1 = ‘difficult to learn’, 100 = ‘easy to learn’). Respondents expressed a high degree of comfort with applying their newly acquired GIS knowledge with an average score of 79.2 (SD = 18.4) on a scale of 1–100, with 1 = “not comfortable at all and 100 = ‘extremely comfortable’. All respondents said they were very likely to recommend this course to their peers.

On the fifth day of the workshop (Table 1), students had the opportunity to work on mini projects that applied GIS techniques to public data. One of the mini projects utilized publicly available national surveillance data, which included all 10,085 confirmed cases of diphtheria diagnosed in Kano State between April 2022 and December 2023. The data were analysed using QGIS-LTR Version 3.34.11 and presented through choropleth maps, heat maps and hotspot analyses. The students demonstrated the clustering of diphtheria cases and health facilities within the eight metropolitan local government areas of the state. An important finding from that mini project was that the local government area with fewer health facilities had the highest number of diphtheria cases.

**Figure 1.**
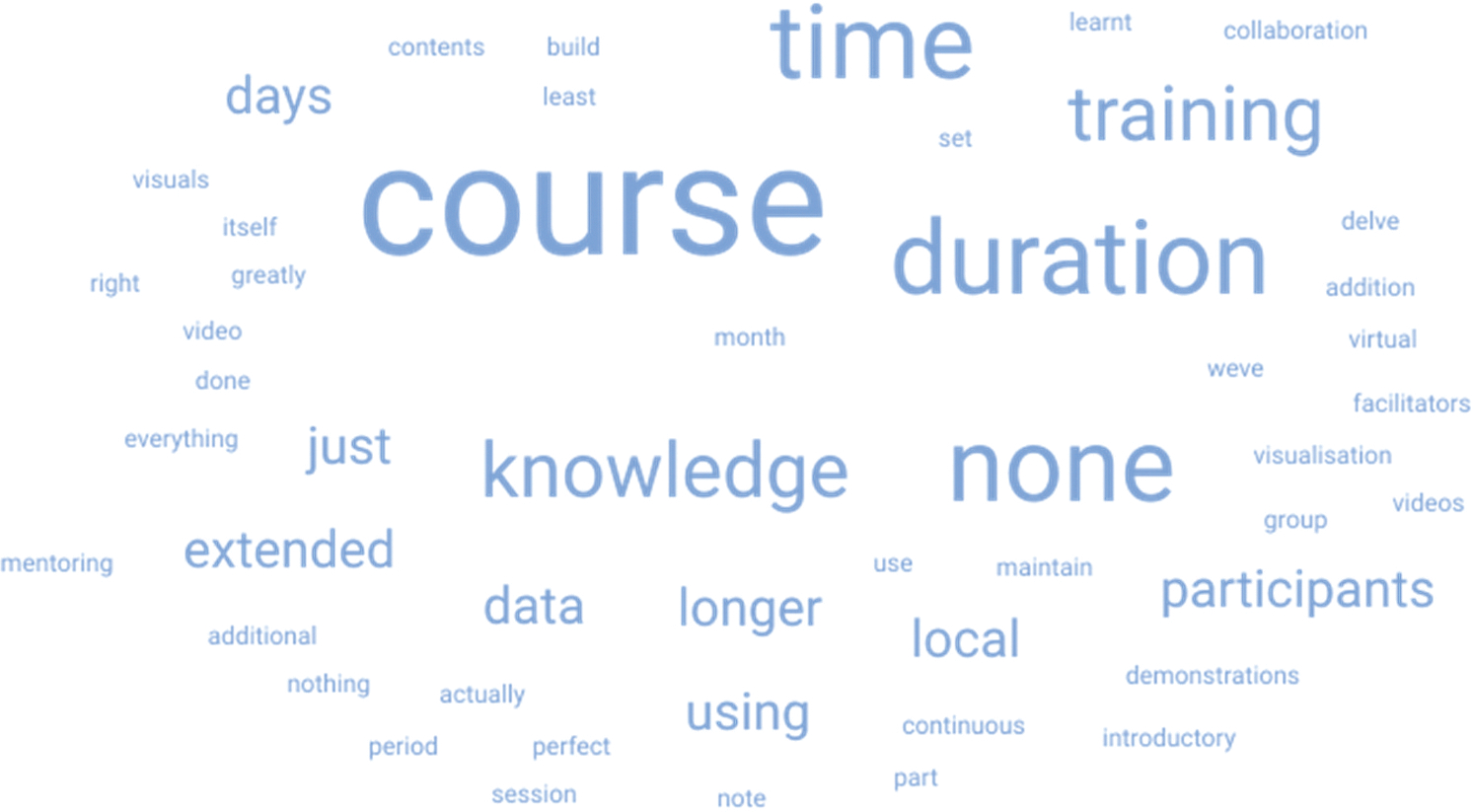
Word cloud representing the frequency of terms used by the respondents when asked about what improvements they would recommend to the course.

Open-ended feedback from the post-workshop survey showed that respondents valued the practical, hands-on activities for applying concepts in real-time (expressed by 13 (45%) of 29 respondents). The most common recommendations for course improvement cited by respondents included allowing more time/extending the duration of the workshop to allow for deeper learning and better retention (9/29, 31.0%), using local data in exercises for more relevant and meaningful training (2/29, 6.9%) and employing more visual aids, like videos and slides, to enhance comprehension of concepts (2/29, 6.9%) (Figure 1). Respondents suggested that future workshops should include topics on advanced GIS, spatial epidemiology, social network analysis, geo-visualization and statistical modelling for infectious disease outbreaks.

## 4. Discussion

Our 5-day intensive GIS workshop targeting physician scientists in northern Nigeria significantly enhanced participants’ knowledge and confidence in GIS-related topics. We found notable improvements across all knowledge areas, with the most significant gains observed in geocoding public health data, use of area-based measures and advanced data visualization. Participants also reported increased confidence in applying GIS tools, particularly in importing spatial data into QGIS, navigating the QGIS interface, mapping public health data into GIS and identifying appropriate GIS software for their projects. The target population for this training consisted of physician scientists based at our local partner institution. The higher average pre-workshop knowledge and confidence scores for visualizing health data likely reflect the clinical background of the respondents.

The workshop syllabus was developed in close collaboration with in-country Nigerian researchers and AKTH/BUK institutional leadership, and therefore reflects the local training needs of participants. We believe that this engagement of institutional leadership and participants fosters meaningful learning experiences for participants and supports the development of future locally relevant and responsive GIS research initiatives. Our use of a learner-adaptive model adjusting and tailoring learning content to the needs of participants also likely improved our ability to improve learning outcomes (Fraulini et al. 2024). Through the mini projects, the students showcased their ability to apply GIS techniques to analyse public health data, including data analysis with QGIS-LTR, spatial visualization and the identification of significant disease patterns and resource distribution. Another strength of this study is our ability to capture the short-term impact of the training on participants’ knowledge and confidence in selected GIS topics and competency areas using pre- and post-workshop surveys. A scoping review of GIS usability, perception and preferences of public health professionals recommended that novice users be trained extensively and provided with regular information technology support and maintenance (Ben Ramadan, Jackson-Thompson, and Boren 2017). We will therefore survey participants at 12- and 24-months post-workshop to ascertain longer term sustainability of the impact of the workshop on participants’ GIS knowledge and skills.

A limitation of this study is that the workshop was conducted only once and lacked a control group who did not attend the training for comparison. In addition, our findings have limited generalizability since participants were drawn from a small group of early-career physician scientists from a single academic medical centre in Nigeria. However, we had representation from different medical disciplines, increasing the relevance of the workshop as a scalable training platform for early-career investigators from a variety of medical disciplines. Another limitation is our reliance on average values to summarize gains in knowledge and competency. These group averages may not accurately reflect the individual-level changes in participants’ knowledge and skills. Finally, the absence of internal consistency and construct validity assessments restricts our ability to evaluate the validity and generalizability of the survey questions used.

In summary, we observed significant improvements across all tested GIS knowledge areas among physician scientists without prior GIS experience, especially in geocoding public health data, using area-based measures, and advanced data visualization. Participants also reported increased confidence in applying GIS tools, particularly in importing and mapping spatial data in QGIS, navigating its interface, and selecting appropriate GIS software for their projects. Participant feedback emphasized the value of hands-on, practical activities and suggested extending the workshop duration, including visual aids and videos, adding more practical sessions and covering advanced GIS techniques and statistical modelling. Incorporating these suggestions into future training would enhance the impact of the foundational knowledge and skills learned in this workshop. Overall, the workshop was highly effective, with participants expressing greater knowledge and competence to apply their new skills in real-world settings. Findings from this interactive capacity-building initiative suggest that similar training opportunities can be beneficial in building GIS knowledge and skills of physician scientists working in similar low- and middle-income settings.

## Figures and Tables

**Table 1.** GIS workshop content/activities by day of workshop, Kano, Nigeria.

Time	Activity/content

**Day 1**	
9:00 a.m. – 9:30 a.m.	Introductions
9:30 a.m. – 5:00 p.m.	GI Science and Spatial Epidemiology
	Topics: GIS Overview, Added Value of GIS and Spatial Epidemiology; Types of GIS Software; Overview of QGIS Software and its features.
	Lab activity: Installing QGIS.
**Day 2**	
9:00 a.m. – 9:40 a.m.	Overview of preceding day sessions
9:40 a.m. – 1:00 p.m.	Fundamentals of Spatial Data I
	Topics: Data Representation and Structure; Map Scale and Projections; Map Types
	Lab activity: Visualizing Health Data
2:00 p.m. – 5:00 p.m.	Geospatial Data Acquisition and Manipulation
	Topics: Preparing Health Event Data II; Geocoding Public Health Data; Area-Based Measures
	Lab activity: Geocoding Health Event Data
**Day 3**	
9:00 a.m. – 9:40 a.m.	Overview of preceding day sessions
9:40 a.m. – 5:00 p.m.	Fundamentals of Spatial Data II
	Topics: Map Scale and Projections; Map types II; Advanced Data Visualization Techniques (Thematic Mapping, Lab Placement)
	Lab activities: Thematic Mapping; Mapping and Map Design
**Day 4**	
9:00 a.m. – 9:40 a.m.	Overview of preceding day sessions
9:40 a.m. – 5:00 p.m.	Geospatial Data Analysis and Modeling I
	Topics: Introduction to Spatial Analysis Concepts and Methods; Performing Basic Spatial Analysis Tasks in QGIS (e.g. Buffer Analysis, Spatial Joins); Descriptive Spatial Statistics; Geoprocessing; Measuring Geographic Distribution
	Lab activities: Geospatial Data Analysis; Descriptive Spatial Statistics
**Day 5**	
9:00 a.m. – 9:40 a.m.	Overview of preceding day sessions
9:40 a.m. – 5:00 p.m.	Practical Applications and Project Work
	Topics: Resources for further learning and career development in GIS and public health
	Lab activities: Participants work on a mini-project applying GIS techniques to public health data; Presentation of mini-projects and feedback
5:00 p.m. – 5:30 p.m.	Closing Ceremony/Certificates

**Table 2.** Demographic characteristics of GIS workshop participants, Kano, Nigeria (*N* = 33).

Characteristic	Number *N* = 33	Percentage (%)

Sex		
Female	10	30.3
Male	23	69.7
Specialty		
Clinical research	1	3.0
Laboratory sciences	6	18.2
Medicine	12	36.4
Pediatrics	2	6.1
Public health	5	15.1
Surgical specialities	7	21.2
Academic rank		
Instructor	4	12.1
Assistant Professor (Senior lecturer)	19	57.6
Associate Professor (Reader)	8	24.2
Other	2	6.0
Prior training in GIScience or Spatial Epidemiology		
Yes	5	15.2
No	28	84.8

Surgical specialities: dentistry/oral maxillofacial surgery, ophthalmology, optometry, otolaryngology/ENT and radiology. Laboratory sciences: chemical pathology, haematology and microbiology. Medicine: cardiology, endocrinology, family medicine, infectious diseases, neurology, nephrology and pulmonology.

Other: research manager, full professor.

**Table 3.** Self-reported average knowledge pre-workshop vs. post-workshop for GIS topic areas and percentage increase in knowledge by topic area, Vanderbilt-Nigeria Building Capacity in HIV/NCDs (V-BRCH) workshop, Kano, Nigeria.

Topic area	Pre-workshop average knowledge*	Post-workshop average knowledge*	Gap	Increase in knowledge %

Spatial epidemiology	42	84	42	100.0
Visualizing health data	48	86	38	79.2
QGIS software	37	87	50	135.1
Spatial data representation and structure	39	84	45	115.4
Map scale and projections	40	82	52	105.0
Geocoding public health data	33	82	49	148.5
Area-based measures	34	78	44	129.4
Advanced data visualization	36	82	46	127.8
Geospatial data analysis	36	78	42	116.7

* Based on a scale of 0–100.

**Table 4.** Self-reported average confidence pre-workshop vs. post-workshop for specific GIS competency areas and percentage increase in confidence by topic area, Vanderbilt-Nigeria Building Capacity in HIV/NCDs (V-BRCH) GIS workshop, Kano, Nigeria.

Competency/skill	Pre-workshop average confidence*	Post-workshop average confidence*	Gap	Increase in confidence, %

Identifying appropriate GIS software	36	89	53	147.2
Navigating QGIS interface	36	90	54	150.0
Importing spatial data into QGIS	36	91	55	152.8
Visualizing health data	42	88	46	109.5
Geocoding health event data	34	80	46	135.3
Conducting thematic mapping	34	79	45	132.3
Mapping public health data in QGIS	34	85	51	150.0
Conducting geospatial data analysis	35	76	41	117.1
Generating descriptive spatial statistics	36	78	42	116.7

* Based on a scale of 0–100.

**Table 5.** Self-reported post-workshop course and instructor evaluation results, Vanderbilt-Nigeria building capacity in HIV/NCDs (V-BRCH) GIS workshop, Kano, Nigeria.

Course item	*N* = 29

**Difficulty of the course**	
Mean	47.1
Standard Deviation	23.4
**Organization of the course**	
Mean	84.6
Standard Deviation	18.1
**Ease of learning information**	
Mean	79.0
Standard Deviation	16.2
**Level of comfort in putting GIS knowledge into practice**	
Mean	79.2
Standard Deviation	18.4
**Overall rating of the course**	4.7/5
**Effectiveness of instructors**	4.9/5
**Likelihood of recommending the course to other clinical researchers**	
‘Very Likely’	100%
